# Translation, cross-cultural adaptation, and validation of the Behavioral Activity Rating Scale (BARS) for the Brazilian population

**DOI:** 10.47626/2237-6089-2021-0310

**Published:** 2023-01-04

**Authors:** Lucas Alves Pereira, Antônio Geraldo da Silva, Curt Hemanny, Rogério de Jesus, Maíra Moromizato, Túlio Vieira, Murilo Souza, Manuela Garcia Lima, Leonardo Baldaçara

**Affiliations:** 1 Centro Universitário UNIFTC Salvador BA Brazil Centro Universitário UNIFTC, Salvador, BA, Brazil.; 2 Faculdade de Medicina Escola Bahiana de Medicina e Saúde Pública Salvador BA Brazil Faculdade de Medicina, Escola Bahiana de Medicina e Saúde Pública, Salvador, BA, Brazil.; 3 Associação Brasileira de Psiquiatria Rio de Janeiro RJ Brazil Associação Brasileira de Psiquiatria, Rio de Janeiro, RJ, Brazil.; 4 Faculdade de Medicina Universidade do Porto Conselho Federal de Medicina Porto Portugal Faculdade de Medicina, Universidade do Porto, Conselho Federal de Medicina, Porto, Portugal.; 5 Faculdades Integradas Padrão Guanambi BA Brazil Faculdades Integradas Padrão (FIP), Guanambi, BA, Brazil.; 6 União Metropolitana de Educação e Cultura Lauro de Freiras BA Brazil União Metropolitana de Educação e Cultura (UNIME), Lauro de Freiras, BA, Brazil.; 7 Hospital Juliano Moreira Salvador BA Brazil Hospital Juliano Moreira, Salvador, BA, Brazil.; 8 Comissão de Emergências Psiquiátricas Associação Brasileira de Psiquiatria Rio de Janeiro RJ Brazil Comissão de Emergências Psiquiátricas, Associação Brasileira de Psiquiatria, Rio de Janeiro, RJ, Brazil.; 9 Universidade Federal do Tocantins Palmas TO Brazil Universidade Federal do Tocantins, Palmas, TO, Brazil.

**Keywords:** Psychomotor agitation, sedation, psychiatric emergencies, emergencies, psychometrics

## Abstract

**Introduction:**

Few instruments are available in Brazil to evaluate psychomotor activity in psychiatric emergency, clinical, and research settings. This study aimed to perform a cross-cultural adaptation of the Behavioral Activity Rating Scale (BARS) into Brazilian Portuguese and assess the adapted scale’s psychometric properties.

**Method:**

An expert consensus committee conducted a translation and back-translation of the original scale, resulting in the BARS-BR. Four pairs of physicians administered the BARS-BR and the Sedation-Agitation Scale (SAS) to patients in a hospital psychiatry emergency room and patients in the hospital’s psychiatric wards. The BARS-BR was compared to the SAS to assess concurrent validity and internal consistency was evaluated with the Bland-Altman technique.

**Results:**

In the emergency room, the correlation coefficients between the first and second assessments were rho = 0.997 and rho = 1.0, respectively. In the hospital wards, the correlation coefficient between the pair of evaluators was rho = 0.951. There were strong correlations between the BARS-BR score of the first examiner and the SAS score of the second examiner (rho = 0.903) and between the SAS score of the first examiner and the BARS-BR score of the second examiner (rho = 0.893).

**Conclusion:**

The BARS-BR showed good psychometric properties, and we recommend its use because it constitutes an easy method for assessment of changes in psychomotor activity. Further studies are suggested to evaluate adoption and comprehension of the BARS-BR scale by all classes of healthcare professionals.

## Introduction

A medical emergency is defined as a situation involving imminent risk to life and requiring immediate and unavoidable intervention.^1 , 2^ Psychiatric emergencies entail behavioral changes that result in risk to the patient or to others and require immediate therapeutic intervention (in minutes or a few hours) to prevent harmful effects. The most common psychiatric emergencies include suicidal behavior, depressive episodes, manic episodes, self-mutilation, markedly compromised critical judgment, severe self-neglect, intoxication or withdrawal states, psychomotor agitation, and aggressiveness.^2 - 6^

Psychomotor agitation or abnormal behavioral activity in patients with psychiatric disorders is a frequent phenomenon and constitutes a clinically relevant condition, not only in emergency situations, but also during hospitalization or in outpatient settings.^6 , 7^ There is scant data on the current prevalence of agitation in medical settings in the world and in Brazil. In the United States, more than 1.7 million patients with agitation are seen at medical emergency services^8 , 9^ in urban centers and the prevalence of agitation in emergency departments is 2.6%.^10^ In Europe, the prevalence of agitation in psychiatric emergencies is 4.6%.^11^ Worldwide, agitation accompanied by aggressiveness are present in 2.6 to 52% of the patients seen in psychiatric emergencies.^8^ In Brazil, the estimated prevalence of agitation is 24% of psychiatric emergencies,^12^ although up-to-date data are needed in this field.

Approximately 10% of patients seen in psychiatric emergencies can become agitated and/or violent during the assessment process,^2 , 6^ which shows that psychomotor agitation is a dynamic situation.^6^ Although the literature implies that in most cases violent behavior occurs without warning signs,^13 , 14^ some authors have indicated that episodes of aggressiveness may be associated with specific risk factors and preceded by behavioral warning signs.^15^

The objective of care for patients with psychomotor agitation, with or without aggression, is to protect them and the people around them by adopting attitudes that aim to reassure them.^2 , 6^ The first step is evaluation, during which the physician should perform an initial mental status examination as soon as possible to determine the most likely cause of the condition and to guide preliminary interventions to calm the patient. Verbal intervention or voluntary medication (medication administered with the patient’s consent) is recommended before moving on to other strategies.^16^ When neither option is possible, administration of medication on an involuntarily basis may become necessary. To this end, the concept of rapid tranquilization should be used: calming the patient without excessive sedation, or even without sedation, with fast-acting medications, with the fewest possible side effects.^17 - 19^

Once the patient is calm, a broader psychiatric evaluation can be completed. In this context, caring for the patient in such a situation requires quick decisions and therefore demands training, professional experience, and technical and scientifically based decisions. It is possible to use scales that allow objective and equal assessments across the team that can also be used to follow-up the approach’s effectiveness.^2^

The American Association of Psychiatric Emergencies (AAEP) proposes use of the Behavioral Activity Rating Scale (BARS) for triage and management outside the emergency room^20^ because it is based on clinical observation, measuring the severity of agitated behavior using a single item that describes seven levels of severity (from a state of sedation to a state of agitation), and is easy to apply when assessing the motor activity of individuals with mental disorders.^21^ Thus, we chose the BARS for translation, cross-cultural adaptation, and validation in Brazilian Portuguese. It will be the first scale designed to assess motor activity in patients with primary mental disorders to be validated for Brazilian Portuguese. The importance of this study is that it will result in availability of this instrument adapted for use in clinical practice by psychiatrists in Brazil.

## Methods

### Study design

This study was carried out in two phases: 1) translation and cross-cultural adaptation of the BARS into Brazilian Portuguese (resulting in the BARS-BR) and 2) assessment of the psychometric properties of the BARS-BR, Phase 1 was based on recommendations in the literature on how to translate and validate a scale,^22^ carried out in five steps: translation, synthesis, back-translation, review by a committee of experts, and pre-test.

**Figure 1:**
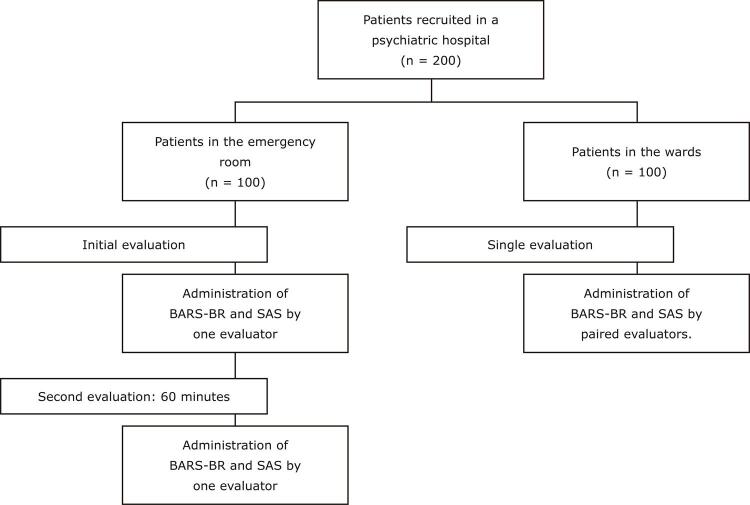
Study flowchart. Eight physicians evaluated the patients in the emergency room individually. Four pairs of physicians evaluated the patients in the wards, at the same time. Each evaluator in the pair applied the scales independently. BARS = Behavioral Activity Rating Scale; SAS = Riker Sedation-Agitation Scale.

Phase 2 consisted of a cross-sectional study. Figure 1 shows a flowchart illustrating this study. Two hundred patients in a psychiatric hospital were divided into two groups and their psychomotor activity was evaluated.

The first group comprised psychiatric patients seen in the emergency room (n = 100). Each patient had their psychomotor activity level evaluated at initial presentation and again after 60 minutes. These two measures were conducted to evaluate administration of the BARS-BR during two phases of psychomotor activity, from agitation at admission to sedation after 60 minutes and with the patient under the effect of treatment. Psychomotor agitation is a common reason for presentation at psychiatric emergency services. These repeated measures enable further evaluation and offer the statistical power to conduct correlation analyses of test-retest and intra-rater comparisons. The second group was composed of patients in the hospital’s male and female wards (n = 100). Each patient in this group was evaluated at a single point in time. These evaluations assessed patients at different levels of agitation or sedation, since they were already receiving intensive psychiatric treatment and had various physical and psychiatric conditions. The data were collected in the emergency room and psychiatric wards of the Hospital Juliano Moreira, a psychiatric referral hospital in the city of Salvador, state of Bahia, Brazil.

To estimate sample size, it is recommended that ten participants should be analyzed per item on a scale.^23^ Since the BARS has one item scored from 1 to 7, we decided to recruit at least 70 participants. We had access to a large sample in a psychiatric hospital and so we were able to assess 200 patients.

This study was evaluated by the research ethics committee at the Escola Bahiana de Medicina e Saúde Pública (EBMSP) (CAAE: 98941018.5.0000.5544) and authorized by one of the authors of the original scale, Joseph Cappelleri.

### Eligibility criteria

The patients included in the study were at least 18 years old and agreed to participate in the study. Consent forms were provided after evaluations with the patients or their legal guardians. Individuals in the emergency room with signs of psychomotor agitation were included. In the psychiatric wards, the severity of psychomotor activity was not an inclusion criterion, enabling assessment of patients with several levels of psychomotor activity (from sedation to agitation). Based on the DSM-5^24^ criteria, patients with unstable clinical diseases, diagnoses of organic disorders, anxiety disorders, or personality disorders were excluded.

### Assessments and Instruments

Two instruments were used in this study: the translated version of the BARS (BARS-BR), to assess the level of behavioral agitation, and the Riker Agitation-Sedation Scale (SAS), to assess patients’ behavioral activity and test convergent validity of BARS-BR. Patients in the emergency room were evaluated at two points in time: when first seen at presentation and after 60 minutes. Patients in the psychiatric wards were evaluated once.

#### Behavioral Activity Rating Scale (BARS)

The BARS^21^ comprises a single item that assesses psychomotor activity across seven intensity categories ranging from score 1 (difficult or unable to rouse) to score 7 (violent, requires restraint). The scale allows evaluation of states ranging from profound sedation to severe agitation. The scale is simple and intuitive and the physician can assess behavioral activity in seconds. The BARS-BR was used to evaluate patients in the emergency room and in the wards.

#### The Riker Sedation-Agitation Scale (SAS)

The SAS^25^ is a single item scale with seven progressive points of severity ranging from agitation (7 = dangerous agitation) to sedation (1 = unarousable).^25 , 26^ The scale is the most compatible scale for evaluation of psychomotor activity available in Brazilian Portuguese^27^ and it was therefore the best choice to compare with the BARS-BR to assess convergent validity.

## Procedures

### Step 1: Translation and cross-cultural adaptation of the BARS into Brazilian Portuguese

Based on the work of Beaton et al.,^22^ adaptation of the scale was accomplished with the following steps: 1) translation; 2) synthesis; 3) back-translation and synthesis; 4) review by a committee of experts; and 5) pre-test.

In step 1 (translation), the English scale was translated into Brazilian Portuguese by two native English teachers. In step 2 (synthesis), the two translators produced a consensus version, resulting in synthesis. In step 3 (back-translation and synthesis), two back-translations were produced by a Brazilian psychiatrist, fluent in English and experienced in psychiatric emergencies, and a native English teacher and professional translator, who then produced a consensus back-translation. Step 4 (review by a committee of experts) involved review of the translations and back-translations by a committee of five experts, each a resident of one of the five regions in Brazil, minimizing cultural distortion.^22^ All five experts were psychiatrists, members of the Comissão de Emergências Psiquiátricas, Associação Brasileira de Psiquiatria, and together they produced a consensus translation of the BARS. The experts had access to all the translations produced up to this point. In step 5 (pre-test), a pre-test of the translation was conducted with a convenience sample (n = 20). Eight evaluators assessed 10 patients in the emergency room and 10 patients in the psychiatric wards using the BARS. The principal investigator (LAP) trained these eight physicians.

The physicians were allocated to pairs, which simultaneously evaluated ten patients in the emergency room and then ten patients in the wards. After the evaluations, each evaluating physician gave a rating between 1 and 10, indicating how easy they found it to understand the scale. The physicians also discussed the application and the scale with the principal investigator; agreeing that no changes were required. Finally, translation distortions were discussed, resulting in a final version of the scale in Brazilian Portuguese called BARS-BR (shown in Table 1 ), ready for assessment of its psychometric properties.

**Table 1:** The Brazilian Portuguese version of Behavioral Activity Rating Scale (BARS-BR)

1.	*Difícil ou incapaz de despertar*
2.	*Adormecido, porém responde normalmente ao contato verbal ou físico*
3.	*Sonolento, parece sedado*
4.	*Calmo e desperto (nível de atividade normal)*
5.	*Sinais de agitação (física ou verbal) aparente, acalma-se sob instruções*
6.	*Extremamente ou continuamente agitado, não requer contenção física*
7.	*Violento, requer contenção física*

### Step 2: Psychometric properties of the BARS-BR

Psychomotor activity was evaluated in 200 individuals, an emergency room group (n = 100) and a psychiatric ward group (n = 100). Before beginning evaluations, the lead researcher (LAP) met with the eight evaluating physicians, explained the study objectives to them, and trained them to apply the BARS-BR and the SAS. These trained physicians evaluated both groups of patients with the BARS-BR.

In group 1, patients in the emergency room (n = 100) were evaluated by 10 psychiatrists. Each psychiatrist individually administered the BARS-BR and the SAS at the beginning of the visit and again 60 minutes after.

In group 2, patients in the wards (n = 100) were evaluated once by a pair of physicians (Evaluator 1 and Evaluator 2) at the same time. Evaluator 1 and Evaluator 2 each administered the BARS-BR and SAS to the same patient. Each of these physicians administered the BARS-BR and the SAS independently in the men’s ward (n = 50) and the women’s ward (n = 50).

In both of the groups, we assessed the internal consistency of the BARS-BR and tested its concurrent validity by evaluating its correlation with the SAS. We used the results of simultaneous administrations of the BARS-BR by different examiners to test inter-rater agreement (between the results of BARS-BR administered by Evaluator 1 and SAS administered by Evaluator 2 and between BARS-BR administered by Evaluator 2 and SAS administered by Evaluator 1).

## Statistical analysis

For validation studies, it is recommended that at least 10 participants are recruited per item on the scale being validated.^28^ A sample size of at least 70 subjects was estimated. Access to a psychiatric referral hospital enabled inclusion of 200 patients. The Statistical Package for the Social Sciences (SPSS)^29^ was used to prepare the database and perform statistical analysis.

Descriptive statistics were extracted, with frequencies, percentages, means, and standard deviations. The content validity index (CVI) was used to assess the proportion of agreement between the expert committee members regarding each of the translated items.^30 , 31^ Each expert rated each of the seven BARS-BR sub-items with a score ranging from 1 (not equivalent) to 4 (absolutely equivalent), and the median score was calculated. The index of agreement must be at least 0.80 and preferably higher than 0.90.^32^

Convergent validity between the BARS-BR and the SAS was evaluated using Spearman’s coefficients (rho) for the correlations between the results of administration of the two scales at the same time. Spearman’s correlation was chosen because we have two continuous, analogue scales with close scores. The variables were obtained by paired observations of participants. Spearman’s correlation allows analysis of data that is not normally distributed, as was the case with our variables. To assess inter-rater reliability, that is, the agreement of scores between the pairs of raters, we analyzed the scores of pairs of physicians who individually evaluated the same patient at the same time in the wards. The evaluators (n = 8) were grouped into pairs for the evaluations conducted in the psychiatric wards (see Figure 1 ).

We used the Bland-Altman (BA) technique^33^ to determine the difference between the BARS-BR and the SAS scores. The BA technique was chosen because both scales evaluate the same construct (psychomotor activity) and they each only have one item rated in the same evaluation (groups 1 and 2). The BA allowed us to identify the variability that occurred between the scale scores and to determine whether this tended to be skewed in any given direction. First, we used a one sample *t* test, where we established whether the variability of these differences differed from 0. Then, we used a bivariate linear regression to verify whether statistical significance existed. The level of statistical significance was set at 0.05.

## Results

### Translation and cross-cultural adaptation of the BARS into Brazilian Portuguese

The translations, back-translations, and consensus versions, along with the original scale, were reviewed by members of a committee of experts. The committee produced an adapted version of the scale and then re-evaluated it. The experts unanimously considered that items 1, 2, 3, and 4 of the version they produced fully retained the literal, semantic, and idiomatic components of the original English version of the BARS. Table 2 shows this process.

**Table 2:** The BARS versions during translation process

Original	Consensus 1: Translation	Consensus 2: Back-translation	Consensus 3: Committee of expert
BARS	Escala de Classificação da Atividade Comportamental	Behavior Activity Classification Scale	Escala de Avaliação da Atividade Psicomotora
1. Difficult or unable to rouse	*Difícil ou incapaz de despertar /acordar*	Difficulty or inability to wake up/stay up	*Difícil ou incapaz de despertar*
2. Asleep, but responds normally to verbal or physical contact	*Adormecido, mas responde normalmente ao contato verbal ou físico*	Sleepy, but responds normally to verbal or physical contact	*Adormecido, porém responde normalmente ao contato verbal ou físico*
3. Drowsy, appears sedated	*Sonolento, parece sedado*	Drowsy, looks sedated	*Sonolento, parece sedado*
4. Quiet and awake (normal level of activity)	*Calmo e desperto (nível de atividade normal)*	Calm and awake (normal activity level)	*Calmo e desperto (nível de atividade normal)*
5. Signs of overt (physical or verbal) activity, calms down with instruction	*Sinais de atividade (física ou verbal) aparente, acalma-se sob instruções*	Visible signs of activity (verbal or physical), calms down under instructions	*Sinais de agitação (física ou verbal) aparente, acalma-se sob instruções*
6. Extremely or continuously active, not requiring restraint	*Extremamente ou continuamente ativo, não requerendo/exigindo restrição*	Extremely or continuously active, no requiring / demanding any restriction	*Extremamente ou continuamente agitado, não requer contenção física*
7. Violent, requires restraint	*Violento, requer/exige restrição*	Violent, requires restraint	*Violento, requer contenção física*

BARS = Behavioral Activity Rating Scale.

During re-evaluation of the initial product, the main issues discussed by the experts were as follows: item 5 of the original version included the phrase “calms down with instruction”; one of the experts suggested that the best translation of this passage would be “calms down with prompting.” The other experts, however, considered the translation “calms down under instruction” to be closer to the original scale, and it was chosen as the final version. According to them, use of the term “instruction” would not cause literal or semantic impairment to understanding of the scale.

In items 6 and 7, the term “restraint” was translated as “physical restraint,” the term most used in Brazilian literature, although the phrase “mechanical restraint” is widely employed. The literal translation would be “restraint,” which would certainly hinder understanding by those administering the instrument. In items 6 and 7, the verb “requires” maintained semantic and idiomatic equivalence to the original scale and was kept by the committee, despite some differences observed during translation. This led to the final version produced by the committee of experts.

### CVI of the cross-cultural adaptation

The CVI score for the seven items of the final version was equal to 1.0, which demonstrates an excellent level of agreement among the members of the committee of experts. There was no need to revise or delete any items.

We concluded that the translated BARS scale presents evidence of content validity, according to the original scale. With the aforementioned adaptations and after evaluation of the CVI of the questions, we proceeded to the next phase regarding the translated and adapted BARS scale, in order to assess the considerations of the evaluating participants.

### Pre-test phase

In the pre-test phase, inter-rater correlations were high in the emergency room (Spearman’s rho = 0.872; p = 0.001), in the psychiatric wards (Spearman’s rho = 1.0; p < 0.0005), and overall (Spearman’s rho = 0.933; p = < 0.0005). These correlations demonstrate *a priori* that the translated and adapted version of the BARS maintains the characteristics of the original instrument.

The outcome of the evaluation among the eight evaluators had a mean of 1.25 points, which denotes that the scale is easy to understand.

The evaluators, in consensus, considered the scale to be easy to administer. No difficulties in understanding the questions or ambiguities of interpretation were reported. The Evaluators agreed that it is a useful instrument for the purpose for which it is intended, and no corrections or other changes to the original version generated by the committee of experts were suggested. Thus, the cross-cultural validity of the scale, now called BARS – Brazilian Portuguese Version (BARS-BR), was established.

### Psychometric properties of the BARS-BR

We assessed a total of 200 patients in the hospital’s emergency room and psychiatric wards. Tables 3 and 4 show the sociodemographic and clinical characteristics of the sample.

**Table 3:** Clinical and demographic characteristics of the sample validation

	Emergency room (n = 100)	Wards (n = 100)
Age, mean (SD)	40.38 (10.70)	35.30 (11.94)
Sex (%)		
Female	44.0	50.0
Male	56.0	50.0
Psychiatric diagnosis* (%)		
Unspecified nonorganic psychosis	33.0	14.0
Bipolar affective disorder	25.0	21.1
Schizophrenia	0.0	23.7
Multiple drug use	0.0	10.5
Disorders due to use of alcohol	8.0	4.0
Dissociative disorders	4.0	0.0
Specific personality disorders	3.0	2.0
Mental retardations	2.0	9.3
Others	1.0	< 5.0

SD = standard deviation.

* Psychiatric diagnoses according to International Statistical Classification of Diseases, 10th revision.

**Table 4:** BARS-BR and SAS scores in the emergency and the wards

	Emergency room (n = 100)		Wards (n = 100)

	Baseline Mean (SD)	Post 60 minutes Mean (SD)	Single evaluation* Mean (SD)
BARS-BR	5.49 (0.77)	3.67 (0.81)	4.23 (1.13)
SAS	5.46 (0.73)	3.70 (0.90)	4.22 (1.13)

BARS-BR = Behavioral Activity Rating Scale – Brazilian Portuguese Version; SAS = Riker Sedation-Agitation Scale; SD = standard deviation.

* This evaluation was performed at a single point in time.

### Convergent validity

In the initial emergency room evaluation (n = 100), conducted individually by eight physicians, there was an excellent correlation between the BARS-BR and the SAS (Spearman’s rho = 0.994; p < 0.0005). In the second evaluation, 60 minutes after the initial evaluation, there was perfect convergent validity between the two scales (Spearman’s rho = 1.0; p < 0.0005).

In the initial assessment, the BARS-BR scores were as follows: item 5 = 68%, item 6 = 15%, and item 7 = 17%. Hence, it was possible to assess the items corresponding to agitated behavior. In the second assessment, item 4 dominated, with 67%, which signals unchanged motor activity; however, sedation categories were also endorsed.

In the assessments of the psychiatric wards sample (n = 100), the pairs of raters selected the same BARS-BR score in 90% of cases. There was a strong inter-rater correlation between the BARS-BR scores recorded by each member of the pairs (Spearman’s rho = 0.951; p < 0.0005). A 90% match was observed between the BARS-BR option selected by rater 1 and the corresponding SAS item selected by rater 2, and there was an 89% match between the SAS option selected by rater 1 and the BARS-BR option selected by rater 2.

In the wards, we analyzed the inter-rater correlations between the BARS-BR and the SAS conducted by the pairs of evaluators. Inter-rater correlations were strong between the BARS-BR administered by Evaluator 1 and the SAS administered by Evaluator 2 (Spearman’s rho = 0.903; p < 0.0005) and vice-versa: BARS-BR administered by Evaluator 2 and the SAS administered by Evaluator 1 (Spearman’s rho = 0.893; p < 0.0005). Evaluator 1 had high intra-rater correlations for BARS-BR (Spearman’s rho = 0.930; p < 0.0005) and for the SAS (Spearman’s rho = 0.923; p < 0.0005). Table 5 shows Spearman’s coefficients in the main correlations.

**Table 5:** Intra and inter-rater Spearman’s correlation coefficients (rho) for the assessments in the wards

	Intra-rater Spearman’s correlation		Inter-rater Spearman’s correlation	

	BARS-1	SAS-1	BARS-2	SAS-2
BARS-1	-	0.930*	0.951*	0.903*
SAS-1	0.930*	-	0.893*	0.972*
BARS-2	0.951*	0.893*	-	0.923*
SAS-2	0.903*	0.972*	0.923*	-

Numbers 1 and 2 correspond to each of the two evaluators.

BARS-BR = Behavioral Activity Rating Scale – Brazilian Portuguese Version; SAS = Riker Sedation-Agitation Scale.

* p < 0.0005.

### Differences between the BARS-BR and SAS

We used the BA technique to determine the difference between the scores generated in the evaluations where both scales were employed; we placed them on a regression graph as being dependent on the mean obtained from the two scales. Agreement between the scales was analyzed for 300 assessments. In the initial emergency room assessment (n = 100) and the final assessment (n = 100), the same rater’s scores were utilized. In the wards, the BARS-BR scores from the first rater were compared to the SAS scores from the second rater (n = 100), evaluating the 300 scenarios where the scales were applied simultaneously.

**Figure 2:**
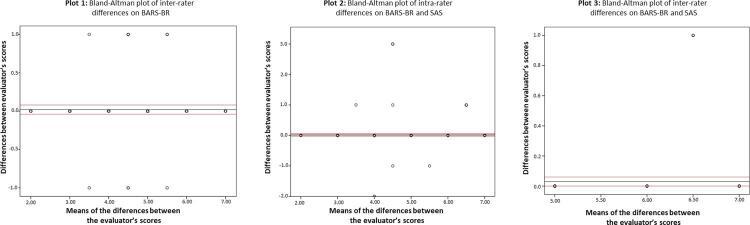
Bland-Altman plots. Plot 1 shows differences between Behavioral Activity Rating Scale – Brazilian Portuguese Version (BARS-BR) scores according to different evaluators. Plot 2 shows differences between BARS-BR and Riker Sedation-Agitation Scale (SAS) according to the same evaluator at different times. Plot 3 shows differences between BARS-BR and SAS scores in the 300 evaluations conducted at the same time.

The line of difference between the scales was close to 0, as shown in Figure 2 , demonstrating no deviation trend. Thus, we can conclude that correlations were strong for the concurrent validity and reliability of the BARS-BR.

## Discussion

As part of the cross-cultural adaptation,^22 , 34^ after translation and evaluation of the instrument by the committee of experts, a convenience sample of psychiatrists evaluated the BARS^25^ from the perspective of comprehensibility. The final version, the BARS-BR, was the outcome of this work, incorporating the final corrections made by all experts and evaluators and shows good psychometric properties.

Evidence of concurrent validity was demonstrated by comparing the scores for the two scales when administered simultaneously by a single rater, by comparing BARS-BR scores from two different raters, and by comparing BARS-BR scores with SAS scores from different raters, in common with other studies.^27 , 35^ Inter-rater reliability was observed across a broad spectrum of patients, demonstrated by the excellent degree of agreement between evaluations.^36^

The BARS-BR is a simple-to-use instrument for assessment of motor activity in psychiatric patients.^21^ It has well-defined criteria and enough levels to assess initial motor activity, as well as to monitor response to pharmacological therapies used in the management of psychomotor agitation. Although two different conditions (agitation and sedation) are assessed on a single scale, the sequential approach establishes a single score, appraising agitation first and then sedation.

The BARS-BR is an excellent option for a wide range of uses in different clinical settings, because it is a single-item scale with seven levels of ascending severity ranging from sedation to agitation.^25^ The scale can also be administered after a few minutes of training. This allows professionals to evaluate patients without interfering with their routine or time. The BARS-BR scores can be easily understood by the hospital staff and it is the first scale validated in Brazilian Portuguese for use in psychiatric emergency settings.^27^ The BARS-BR can be used in clinical trials to evaluate the effect of psychopharmacological and non-pharmacological treatments.

The purpose of validating the scale was to establish a clinically useful tool for evaluating agitated behavior and sedation levels in psychiatric patients in a hospital setting. In theory, use of this scale could improve communication among mental health staff and their approach to patients with psychomotor agitation by standardizing the description of patients’ psychomotor behavior, commonly measured with non-technical terms that may give rise to misinterpretation. Thus, we emphasize the ease of use and clarity of the psychometric instrument in question.

### Limitations and perspectives

The validation of any scale is an evolving process. Since we did not have fully bilingual raters, it was not possible to comparatively evaluate the original BARS scale and the BARS-BR. Thus, the evaluation of the instrument’s internal consistency may be somewhat compromised. The committee of experts, however, was made up of bilingual psychiatrists. We can infer that there was no compromise of cultural aspects in the adaptation process and that semantic equivalence with the original version was maintained.

In the validation study of the original scale,^21^ the instrument’s stability was evaluated by determining the consistency in scores when the same set of raters saw the same six clinical vignettes on two occasions, with an interval of approximately 8 months. Therefore, the consistency of repeated measures was not evaluated in the present study.

In this study, the number of patients who had sedation scores was considerably lower than the number of agitated patients. There was, however, an important agreement between BARS-BR and SAS in evaluation of subjects who had sedation scores. Considering that the SAS was originally configured for use in the intensive care setting, where the prevalence of sedated patients is possibly higher than in psychiatric units, we can deduce that the BARS-BR is useful for assessing individuals with slowed motor activity.

A proportion of the participants showed normal behavioral activity at baseline, according to the BARS-BR, with a score of four. This may reflect the fact that psychomotor activity, although critical, is only one of the multiple dimensions that characterize the overall profile of patients with mental disorders, so one might consider that psychomotor activity is only one aspect of agitation.

One question that may be better elucidated by future investigations is the ability of the BARS-BR (vs. other existing instruments) to demonstrate differences between agents, in regards to tranquilization or beneficial sedation (vs. excessive sedation). It would also be important to investigate the performance of the BARS-BR for assessing individuals who were too agitated to give written informed consent for their inclusion in this study. Such limitations also occurred in the validation studies of the original BARS scale.

We believe that the factors mentioned above did not negatively impact assessment of this instrument’s psychometric properties. We suggest, however, that future studies verify understanding of the scale by other professionals who use it, since they will in fact have contact with the written material of the scale. Overall, there is no evidence against the adequacy of the translation and adaptation of the BARS for validation of the Brazilian version.

## Conclusion

The present study presents evidence that the translation and cross-cultural adaptation of the BARS were satisfactory and successful, considering that content validity and cross-cultural validity were demonstrated for the Brazilian Portuguese version. We followed steps suggested in the literature^22^ to make the BARS-BR possible.

We found significant correlations between the scores derived from this scale and the SAS scale, supporting these theoretical positions. Hence, this psychometric instrument is capable of measuring motor activity in acute psychiatric patients consistently across time, raters, and items. Additional studies are needed to fill certain methodological gaps, however, in order to make a categorical statement.

This work provides the Brazilian population with an easily applicable scale, capable of assessing initial changes in psychomotor behavior and sensitive to changes related to pharmacological treatment, as observed in validation studies of the original scale. Based on the present study, the BARS-BR may be used in formulation of protocols for the care of patients with altered motor activity secondary to mental illness, thus providing individualized and effective management in this context.
